# Performance of a HRP-2/pLDH based rapid diagnostic test at the Bangladesh-India-Myanmar border areas for diagnosis of clinical malaria

**DOI:** 10.1186/1475-2875-12-378

**Published:** 2013-10-30

**Authors:** Rubayet Elahi, Abu Naser Mohon, Wasif A Khan, Rashidul Haque, Mohammad Shafiul Alam

**Affiliations:** 1 International Centre for Diarrhoeal Disease Research Bangladesh (icddr,b), Dhaka, Bangladesh; 2 Department of Neurobiology and Developmental Sciences, University of Arkansas for Medical Sciences (UAMS), Little Rock, AR, USA

## Abstract

**Background:**

The rapid diagnostic test (RDT) has been adopted in contemporary malaria control and management programmes around the world as it represents a fast and apt alternative for malaria diagnosis in a resource-limited setting. This study assessed the performance of a HRP-2/pLDH based RDT (Parascreen® Pan/Pf) in a laboratory setting utilizing clinical samples obtained from the field.

**Methods:**

Whole blood samples were obtained from febrile patients referred for malaria diagnosis by clinicians from two different Upazila Health Complexes (UHCs) located near the Bangladesh-India and Bangladesh-Myanmar border where malaria is endemic. RDT was performed on archived samples and sensitivity and specificity evaluated with expert microscopy (EM) and quantitative PCR (qPCR).

**Results:**

A total of 327 clinical samples were made available for the study, of which 153 were *Plasmodium falciparum-*positive and 54 were *Plasmodium vivax-*positive. In comparison with EM, for *P. falciparum* malaria, the RDT had sensitivity: 96.0% (95% CI, 91.2-98.3) and specificity: 98.2% (95% CI, 94.6-99.5) and for *P. vivax*, sensitivity: 90.7% (95% CI, 78.9-96.5) and specificity: 98.9% (95% CI, 96.5-99.7). Comparison with qPCR showed, for *P. falciparum* malaria, sensitivity: 95.4% (95% CI, 90.5-98.0) and specificity: 98.8% (95% CI, 95.4-99.7) and for *P. vivax* malaria, sensitivity: 89.0% (95% CI,77.0-95.4) and specificity: 98.8% (95% CI, 96.5-99.7). Sensitivity varied according to different parasitaemia for falciparum and vivax malaria diagnosis.

**Conclusion:**

*Parascreen® Pan/Pf Rapid test for malaria* showed acceptable sensitivity and specificity in border belt endemic areas of Bangladesh when compared with EM and qPCR.

## Background

Malaria is often lethal with high potential expenditure for health if diagnosis is inaccurate [1]. Accurate diagnosis of malaria is of increasing importance as the prevalence of malaria is declining around the globe, making surveillance and screening more important for programme management [2,3] and to restrict the use of anti-malarial drugs to restrain the spread of drug resistance [4].

For decades, expert microscopy (EM) of peripheral thick and thin blood smears has been the standard diagnostic test for malaria, however, it is time consuming and requires substantial expertise [1,5]. Enzyme-linked immunosorbent assay (ELISA) and polymerase chain reaction (PCR)-based diagnostic tests have been introduced which ameliorate sensitivity and specificity of malaria diagnosis, but only in reference settings where well equipped laboratory facilities are available, making it difficult to implement in a field setting [6]. Other nascent molecular methods, such as loop-mediated isothermal amplification (LAMP) [7-9] and real-time quantitative nucleic acid sequence-based amplification (QT-NASBA) [10] are in use, but the efficacy of each is unproven.

After being introduced in the early 1990s, rapid diagnostic tests (RDTs) have become an attractive alternative to the above-mentioned methods in a resource-limited setting for malaria diagnostics. The antigen-based RDTs detect specific antigens produced by malaria parasites by reaction with bound antibodies on an absorbent nitrocellulose membrane. Among several types of RDTs the two-band tests and three-band tests are most widely used. Two-band tests either detect only one species (*Plasmodium falciparum)*, usually by detecting histidine-rich protein 2 (HRP2), or detect any of the four most common malaria parasites (*P. falciparum, Plasmodium vivax, Plasmodium malariae* and *Plasmodium ovale)*, typically by detecting pan-*Plasmodium*-specific lactate dehydrogenase (pLDH), while three-band tests detect both the *P. falciparum*-specific antigen HRP2 and the pLDH or any one species specific LDH (mostly *P. vivax*). The third band is the test control band [1,5,11].

Southeast Bangladesh, northeast India and southwest Myanmar are similar in geographical characteristics and endemic for malaria. *Plasmodium falciparum* is the most abundant parasite, followed by *P. vivax* in these countries [6,11,12]. The presence of *P. malariae* and *P. ovale* has also been reported in each country [13-16]. These three countries share their borders, making trans-border malaria transmission plausible. The presence of all four parasites in these mostly remote and resource-limited areas illustrate the importance of a RDT that can detect all malaria parasites. Amongst the locally available RDTs, *Parascreen® Pan/Pf Rapid test for malaria* (Zephyr Biomedical Systems, India), hereafter noted as Parascreen, is a RDT that has the capability to detect all types of human malaria, as it detects *P. falciparum-*specific HRP-2 and pan-*Plasmodium*-specific LDH. It has been evaluated against microscopy and conventional PCR in field and laboratory settings [12,17-24]. Here, the assessment of Parascreen in a laboratory setting and its performance compared with EM and qPCR are described.

## Methods

### Study area and population

Whole blood samples were obtained from febrile patients with clinical symptoms referred for laboratory investigation between May 2009 to December 2010. The represented regions include Matiranga Upazila in Khagrachari district and Ramu Upazila in Cox’s Bazar district, two different subdistricts of the southeastern part of Bangladesh from corresponding UHC. Matiranga borders Tripura state of India and Ramu borders Myanmar, where malaria is endemic [15,16] and is caused mainly by *P. falciparum* and *P. vivax*.

### Sample collection

An expert medical technologist collected approximately 5 mL of blood from adult subjects and 3 mL from minor subjects by venipuncture. Thick and thin blood films were prepared in duplicate using two drops of blood for each sample. The remaining blood was preserved at -20°C in EDTA tube and transported to the Parasitology Laboratory, icddr,b in cool boxes maintaining the temperature below 4°C using ice bags.

Approval from Research Review Committee (RRC) and Ethical Review Committee (ERC) of icddr,b was obtained for this study. Permission for conducting the study was obtained from the National Malaria Control Programme (NMCP). All participants or legal guardians signed informed consent before participant enrolment and sample collection. Complete anonymity was maintained at each stage of the study.

### Expert microscopy (EM)

Blood smears were stained with Giemsa and screened for parasites under the (100X) oil immersion lens at the field site by experienced microscopists in the corresponding UHC. The microscopy results were confirmed by a second independent, experienced microscopist who was blinded to prior results. Parasite density was determined by both microscopists counting the parasites and leucocytes [25] and the average was used for the study. When there was any disagreement in diagnosis by the two microscopists for any sample and mixed (*P. falciparum* and *P. vivax*) infection were excluded from the study.

### Rapid diagnostic tests (RDTs)

Parascreen (Zephyr Biomedical Systems, India; Lot No 101159) is a three-band antigen detection RDT which comes in cassette format. It employs a recombinant antibody against pLDH to detect *Plasmodium*-specific LDH and anti-HRP2 antibody to detect *P. falciparum*-specific HRP2. All RDTs were performed on archived blood samples by trained and skilled laboratory personnel at the Parasitology Laboratory, icddr,b following the manufacturer’s instructions. Briefly, one pink-purple line in the proximal area (control line) interprets negative for malaria; one pink-purple line in the middle area, along with the control line, interprets non-*P. falciparum* infection, exclusively *P. vivax* in this study; one pink-purple line, along with the previous two bands, interprets *P. falciparum* infection. If any of the two test lines or control line did not appear, the test was invalid and repeated.

### DNA isolation

DNA was isolated using QIAamp DNA blood mini kit (Qiagen Sciences Inc, USA) following the manufacturer’s instructions from 200 μL of archived whole blood.

### qPCR

Quantitative PCR (qPCR) was performed on isolated DNA following the method described by Alam *et al.*[6] with Invitrogen® SYBR Green I supermix UDG (Life Technologies Corporation, USA). The sensitivity and specificity of qPCR for *P. falciparum* was 97.1 and 97.6%, respectively, while for *P. vivax* 95.2 and 98.1% [6]. Any mixed (*P. falciparum* and *P. vivax*) infection diagnosed by qPCR was not considered in this study.

### Data analysis

All data were encoded in an Excel data sheet and the performance of RDT was calculated by means of the following indicators: sensitivity, specificity, positive predictive value (PPV), negative predictive value (NPV) and agreement (kappa) were calculated with their corresponding 95% confidence intervals (95% CI), using EM and qPCR as reference standards. Sensitivity was calculated as the proportion of positive RDT test results among malaria-positive samples identified by EM and qPCR, while specificity was calculated as the proportion of negative test results among the malaria-negative samples identified by the reference standards. PPV and NPV were obtained as the true positive results among all malaria-positive samples and the true negative results among all negative samples, respectively [26]. Agreement (k) analysis was conducted in IBM SPSS Statistics, version 17.0 (IBM Corporation, NY, USA) by creating a 2 × 2 contingency table.

## Results

In total, 327 febrile patients were included in this study from two UHCs. The results of EM, qPCR and Parascreen are provided in Table 1. With EM, there were 207 (63.3%) positive malaria cases, of which 153 (73.9%) were *P. falciparum* infection and 54 (26.0%) were *P. vivax* infection. The parasite density for *P. falciparum* ranged between 16 and 261,480 parasites/μL (IQR: 7,500-50,100) with median value of 19,960 parasites/μL, while the parasite density for *P. vivax* ranged between 16 and 25,120 parasites/μL (IQR: 320–4,800) with median value of 1,140 parasites/μL. qPCR confirmed 208 (63.6%) positive malaria cases, of which 154 (74.0%) were *P. falciparum* and 54 (25.9%) were *P. vivax*. With Parascreen, there were 202 (61.7%) malaria positive cases, of which 150 (74.2%) were *P. falciparum* and 52 (25.7%) were *P. vivax* infection.

**Table 1:** Parascreen® diagnosis results and comparison with diagnosis by EM and qPCR

**Parasreen results**		**Microscopy**			**qPCR**		
		**Negative**	**Pf**	**Pv**	**Negative**	**Pf**	**Pv**
Negative	125	119	3	3	119	3	3
Pf	150	1	147	2	0	148	2
Pv	52	0	3	49	0	3	49
Total	327	120	153	54	119	154	54

Table 2 represents the calculated indicators when Parascreen was compared with EM and qPCR. EM being the reference standard, Parascreen had the following results, for any kind of malaria detection, sensitivity: 97.1% (95% CI, 93.5-98.8) and specificity: 99.1% (95% CI, 96.8-99.9); for *P. falciparum* malaria detection, sensitivity: 96.0% (95% CI, 91.2-98.3) and specificity: 98.2% (95% CI, 94.6-99.5) and for *P. vivax* malaria detection, sensitivity: 90.7% (95% CI, 78.9-96.5) and specificity: 98.9% (95% CI, 96.5-99.7). When qPCR was used as the reference standard, Parascreen had the following results for any kind of malaria detection, sensitivity: 97.1% (95% CI, 93.5-98.8) and specificity: 100% (96.1-100.0); for *P. falciparum* malaria detection, sensitivity: 95.4% (95% CI, 90.5-98.0) and specificity: 98.8% (95% CI, 95.4-99.7) and for *P. vivax* malaria detection, sensitivity: 89.0% (95% CI, 77.0-95.4) and specificity: 98.8% (95% CI, 96.5-99.7).

**Table 2:** Comparative indicators of Parascreen®, when using EM and qPCR as reference standard

**Reference standard**	**Test**	**Results by Parascreen**				
		**Sensitivity [%(95% CI)]**	**Specificity [%(95% CI)]**	**PPV [%(95% CI)]**	**NPV [%(95% CI)]**	**Agreement (k)**
EM	Overall	97.1 (93.5-98.8)	99.1 (94.7-99.9)	99.5 (96.8-99.9)	95.2 (89.4-98.0)	0.954
	Pf	96.0 (91.2-98.3)	98.2 (94.6-99.5)	98.0 (93.8-99.4)	96.6 (92.4-98.6)	0.945
	Pv	90.7 (78.9-96.5)	98.9 (96.5-99.7)	94.2 (83.0-98.4)	98.1 (95.5-99.3)	0.910
qPCR	Overall	97.1 (93.5-98.8)	100.0 (96.1-100.0)	100.0 (97.6-100.0)	95.2 (89.4-98.0)	0.961
	Pf	95.4 (90.5-98.0)	98.8 (95.4-99.7)	98.6 (94.7-99.7)	96.0 (94.7-99.7)	0.945
	Pv	89.0 (77.0-95.4)	98.8 (96.5-99.7)	94.2 (83.0-98.4)	97.8 (95.0-99.1)	0.899

**Figure 1:**
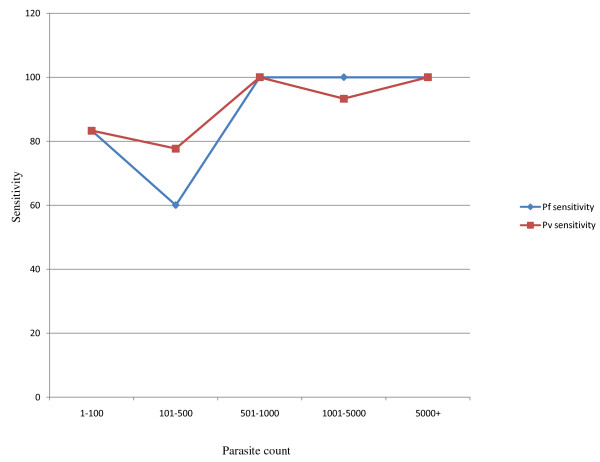
Varied sensitivity of Parascreen® (Pf/pan) according to different parasitaemia.

Parascreen showed higher sensitivity (93.3-100%) in detecting samples with parasite densities >500 parasites/μL for both *P. falciparum* and *P. vivax*, whereas for parasite densities ranging from 1–500 parasites/μL, the sensitivity was low (60.0%-83.3%) (Figure 1).

## Discussion

Parascreen showed acceptable performance in this study with overall sensitivity and specificity of 97.1 and 99.1%, respectively, when compared to EM, and 97.1 and 100%, respectively, in comparison with qPCR. Parascreen can detect all types of non-falciparum malaria but in this study only *P. vivax* was considered as *P. malariae* and *P. ovale* cases were not present in the study samples [13,14]. Parascreen demonstrated varying sensitivity and specificity when compared with EM and qPCR depending on parasite species (*P. falciparum* and *P. vivax)* and parasitaemia of infections.

Several evaluation studies of Parascreen in different countries reported overall sensitivity ranging from 47.5 to 95.5% and specificity from 64.3 to 98.5% with varying performance for falciparum and non-falciparum malaria detection [12,17-21,23,24]. Parascreen has been evaluated by WHO RDT evaluation programme and for *P. falciparum* detection it showed almost 100% detection rates while for *P. vivax* it was approximately 30% [27]. Here, in this study, for *P. falciparum* detection, the sensitivity and specificity was also in concordance with the previous findings [12,20,21], while for *P. vivax* detection, improved sensitivity and specificity are reported. The improved sensitivity and specificity of *P. vivax* detection compared to previous findings may be due to the increased release of antigen through parasite lysis in the archived sample [1] or due to the improvement in the product. This finding is also corroborated by a meta analysis where mean sensitivity and specificity of 95.0 and 95.2%, respectively, for HRP-2 based assays and 93.2 and 98.5%, respectively, for pLDH based assays were calculated [28].

In a study in India, Parascreen showed 94.0% sensitivity and 72.0% specificity for *P. falciparum* and for *P. vivax* 77.2% sensitivity and 98.1% specificity were recorded when compared with EM and similar values observed when compared with PCR [12].

In Myanmar, two RDTs with similar detection properties have been evaluated in field settings [11,26]. The SD 05FK60 RDT evaluated in the Rakhaine state of Myanmar showed 90.2% sensitivity and 98.5% specificity for *P. falciparum* and 79.4% sensitivity and 98.7% specificity for non-falciparum malaria [11]. The VIKIA Malaria Ag Pf/Pan™ test showed 98.0 and 100% sensitivity for *P. falciparum* and non-falciparum malaria, respectively, with specificity of 98.0 and 100%, respectively [26].

*Onsite* (Pf/Pan), a RDT with similar detection principle recently evaluated in Bangladesh, reported 94.2% sensitivity and 99.5% specificity for falciparum malaria detection and for vivax malaria detection it showed sensitivity and specificity of 97.3 and 98.7%, respectively [5] which showed slightly better sensitivity and specificity compared to Parascreen.

WHO recommends sensitivity ≥95% at ≥100 parasites/uL for RDTs [1]. In this study, for both falciparum and vivax malaria detection, sensitivity was less than the recommended values for low parasitaemia; however, considering fewer low parasitaemia samples, statistically valid conclusions have not been attained.

In this study, Parascreen was unable to detect three microscopically confirmed falciparum malaria samples with parasitaemia ranging from 112 to 2,600 parasites/uL. This might be caused by the degradation of HRP-2 target antigen as the study was carried out with archived samples. Intraspecies sequence variation [29], deletions or mutations of HRP-2 gene [30,31] among different *P. falciparum* isolates could also account for false negative tests. The extent of HRP-2 variations in Bangladesh is currently unknown, however variations or deletions in HRP-2 have been reported recently from India, [31] as well as some African countries [30,32]. In this study, three *P. falciparum* samples showed no HRP-2 test line but Pan specific test line, thus considered as *P. vivax*, as other types of malaria were absent in the study samples. The intraspecies variation, mutation or deletions in the HRP-2 gene can cause non-expression of HRP-2 [30] which may explain this. Parascreen identified three *P. vivax*-positive samples with parasitaemia ranging from 16 to 200 parasites/uL as negative. This might be due to low pLDH level, as pLDH level is directly proportional to parasitaemia [33]. In many studies, a reduced sensitivity for non-falciparum malaria detection, compared to falciparum detection, in combined HRP-2/pLDH RDTs has also been reported [5,6,11,26].

As all four malarial parasites co-exist in the Bangladesh-India-Myanmar border area, an important criterion for selection of an appropriate RDT is the capability to detect all types of malaria. It is advantageous to use Pf/Pan RDTs which can do so. The high predictive values for Parascreen indicate that it is able to detect true malaria cases as well as ruling out non-malaria cases. High sensitivity, specificity and predictive values for Parascreen present it as a viable alternative for malaria diagnostics in Bangladesh-India-Myanmar border areas where malaria is endemic.

The absence of *P. malariae* and *P. ovale* samples in this study restricts the findings to the detection performance of falcipaum and vivax malaria. The inclusion of *P. malariae* and *P. ovale* in the study samples is needed to assess non-falciparum malaria detection performance.

## Conclusion

Parascreen showed acceptable performance for falciparum as well as vivax malaria diagnosis in standard experimental conditions. It can be employed in resource-limited settings to diagnose all types of human malaria.

## Competing interests

The authors declare that they have no competing interests.

## Authors’ contributions

RE and MSA conceptualized and designed the study, collected and identified samples, analyzed the data, drafted the manuscript and made final revisions. RE, ANM and MSA did sample analysis. WAK and RH made critical revision of the manuscript. RE and ANM performed laboratory tests. All the authors read the final version of the manuscript and approved.
